# Quercetin from *Polygonum capitatum* Protects against Gastric Inflammation and Apoptosis Associated with *Helicobacter pylori* Infection by Affecting the Levels of p38MAPK, BCL-2 and BAX

**DOI:** 10.3390/molecules22050744

**Published:** 2017-05-06

**Authors:** Shu Zhang, Jian Huang, Xiaoqin Xie, Yun He, Fei Mo, Zhaoxun Luo

**Affiliations:** 1 Department of Clinical Laboratory, School of Medical Laboratory Sciences, Guizhou Medical University, Guiyang 550004, China; shuzhang@126.com (S.Z.); xiaoqin1@126.com (X.X.); yunhe1@126.com (Y.H.); mofeigy@163.com (F.M.); zhaoxunl@163.com (Z.L.); 2 Department of Clinical Biochemistry, Affiliated Hospital of Guizhou Medical University, Guiyang 550004, China

**Keywords:** 38-kD tyrosine phosphorylated protein kinase, apoptosis, *Helicobacter pylori*, *Polygonum capitatum*, proliferation, quercetin

## Abstract

*Helicobacter pylori*-associated gastritis is a major threat to public health and *Polygonum capitatum* (PC) may have beneficial effects on the disease. However, the molecular mechanism remains unknown. Quercetin was isolated from PC and found to be a main bioactive compound. The effects of quercetin on human gastric cancer cells GES-1 were determined by xCELLigence. *H. pylori*-infected mouse models were established. All mice were divided into three groups: control (CG, healthy mice), model (MG, *H. pylori* infection) and quercetin (QG, mouse model treated by quercetin) groups. IL-8 (interleukin-8) levels were detected via enzyme-linked immunosorbent assay (ELISA). Cell cycle and apoptosis were measured by flow cytometry (FCM). Quantitative reverse transcription PCR (qRT-PCR) and Western Blot were used to detect the levels of p38MAPK (38-kD tyrosine phosphorylated protein kinase), apoptosis regulator BCL-2-associated protein X (BAX) and B cell lymphoma gene 2 (BCL-2). The levels of IL-8 were increased by 8.1-fold in a MG group and 4.3-fold in a QG group when compared with a CG group. In a MG group, G0–G1(phases of the cell cycle)% ratio was higher than a CG group while S phase fraction was lower in a model group than in a control group (*p* < 0.01). After quercetin treatment, G0–G1% ratio was lower in a QG group than a MG group while S phase fraction was higher than a MG group (*p* < 0.01). Quercetin treatment reduced the levels of p38MAPK and BAX, and increased the levels of BCL-2 when compared with a MG group (*p* < 0.05). Quercetin regulates the balance of gastric cell proliferation and apoptosis to protect against gastritis. Quercetin protects against gastric inflammation and apoptosis associated with *H. pylori* infection by affecting the levels of p38MAPK, BCL-2 and BAX.

## 1. Introduction

*Helicobacter pylori* is a gram-negative spiral bacterium that colonizes human gastric epithelial cells, causing epithelial cell inflammation, such as gastritis [1,2]. More than 70% gastritis are caused by *H. pylori* infection, which is responsible for more than 90% of active gastritis [3]. *H. pylori* infection is the main cause of chronic gastritis, which is called *H. pylori*-associated gastritis (HAG) [4,5]. *H. pylori* infection causes gastric mucosal layer edema and neutrophil infiltration, leads to acute gastric mucosal inflammation, and then results in chronic gastritis. These lymphocytes and plasma cells, mucosal layer atrophy, intestinal metaplasia and epithelial dysplasia, ultimately lead to the formation of cancer [5,6].

*H. pylori* has been classified as the main gastric cancer carcinogen by World Health Organization [7]. Reducing or eradicating *H. pylori*-induced mucosal inflammation will promote healing of ulcers, reduce the risk of gastric cancer, and delay the process of mucosa-associated lymphoid tissue (MALT) lymphoma [8,9]. However, the most used methods for *H. pylori* eradication require high cost and have significant side effects, including diarrhea [10,11] and gastrointestinal abnormal responses [11]. Meanwhile, the eradication rate is not satisfactory because of drug resistance [12,13]. To address the issue, the development of new drugs for the therapy of *H. pylori* infection has become very urgent.

Chinese herbal medicine has multi-target effects and fewer side effects, and it has been developed in anti-infection therapy [14,15]. Medicine researchers have committed to screen the drugs against *H. pylori* and explore the related mechanism. Cimetidine has been used to treat the patients with *H. pylori*-reduced erosive gastritis and duodenal ulcers. The results show that betel nut clearance rate in cimetidine group is better than a control group [16,17]. However, the drug has low antibacterial activity [18] and further work needs to address the problem. *Polygomun capitutam* (PC), a traditional Miao-nationality medicinal plant belonging to *Persicaria* genus, is mainly distributed in southwest China. The main components of PC extracts are flavonoids, phenolic acids and lignans [19], and glycoside [20]. Previous studies show that extracts of PC have anti-bacterial effects on *Staphylococcus aureus, Staphylococcus epidermidis*, *Escherichia coli* and *Klebsiella pneumoniae* as well as anti-inflammatory [21,22], and anti-oxidative activities [23].

Therefore, we established a *H. pylori*-infected mouse model to explore the molecular mechanism for *H. pylori* infection from cell proliferation and apoptosis. We expect to develop a new anti-*H. pylori* drug by using the quercetin isolated from PC. The increased levels of p38MAPK (38-kD tyrosine phosphorylated protein kinase) will result in the upregulation of *H. pylori* proliferation [24]. Increased expression of anti-apoptotic gene Bcl-2 has also been found in the gastric mucosa infected by *H. pylori* in children [25]. The upregulation of *H. pylori* infection on the pro-apoptotic gene, BCL-2-associated protein X (BAX), is higher than its induction of B cell lymphoma gene 2 (BCL-2), which will result in the apoptosis in patients with chronic gastritis [26]. Thus, the levels of p38MAPK, BCL-2 and BAX are closely related to the development of *H. pylori*-infected gastritis. We explored the effects of quercetin on these molecules.

## 2. Results and Discussion

### 2.1. Characterization of PC

**Figure 1:**
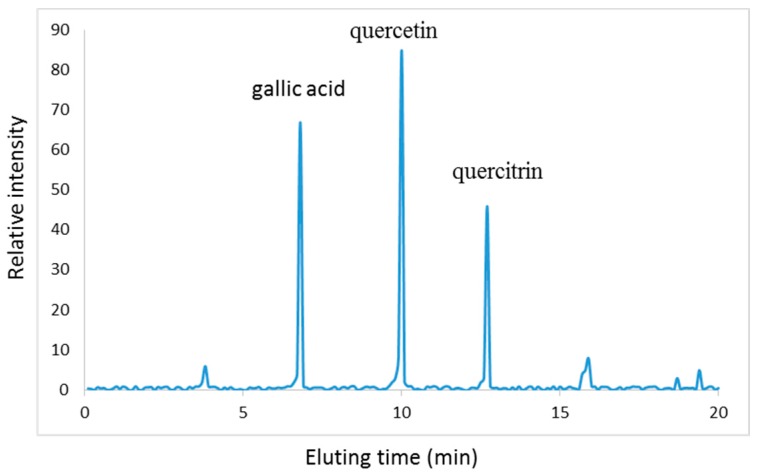
High performance liquid chromatography (HPLC) analysis of main components of *Polygonum capitatum* (PC). Three main components (gallic acid, quercitrin, and quercetin) were isolated from PC after HPLC detection and further confirmed by electrospray ionization mass spectrometry (ESI-MS).

**Figure 2:**
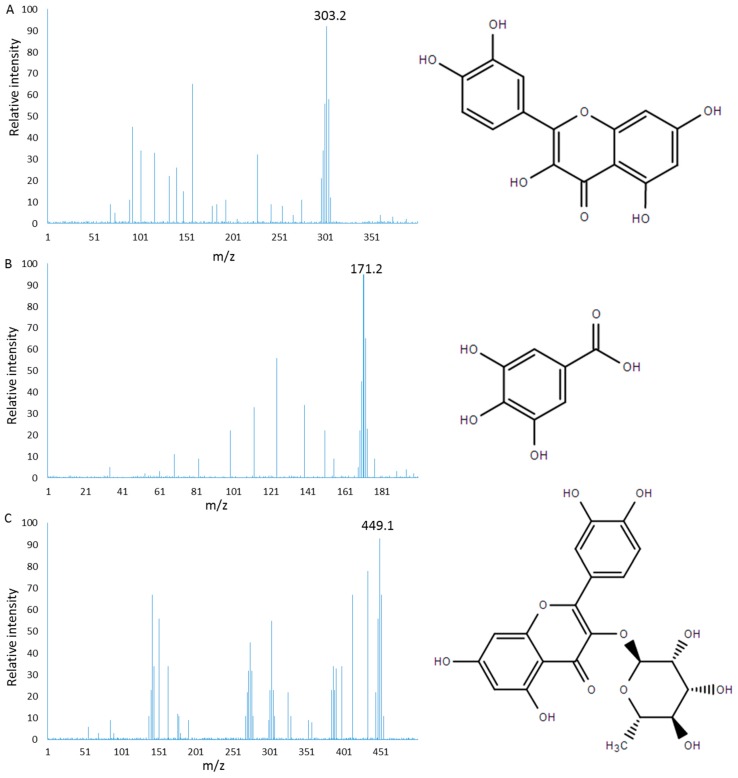
ESI MASS spectrometry analysis of bioactive fractions from PC under the conditions that produced mass spectra with M + H^+^: (**A**) mass spectra visualized following the separation of quercetin (M + H^+^ = 303 Da); (**B**) mass spectra visualized following the separation of gallic acid (M + H^+^ = 171 Da); and (**C**) mass spectra visualized following the separation of quercitrin (M + H^+^ = 449 Da).

As Figure 1 shows, PC is rich in polyphenol, which is beneficial for controlling infection. Three main components (mg/100 g, gallic acid 32, quercitrin 19, and quercetin 53) were isolated from PC after high performance liquid chromatography (HPLC) detection, which were concordant with a report [27]. The mass of each extraction aliquot was further confirmed by electrospray ionization mass spectrometry (ESI-MS) under the conditions that produced mass spectra with M + H^+^. Figure 2 shows that the masses for quercetin (Figure 2A), gallic acid (Figure 2B) and quercitrin (Figure 2C) were 302, 170 and 448 Da, respectively.

### 2.2. GES-1 Growth Curve

**Figure 3:**
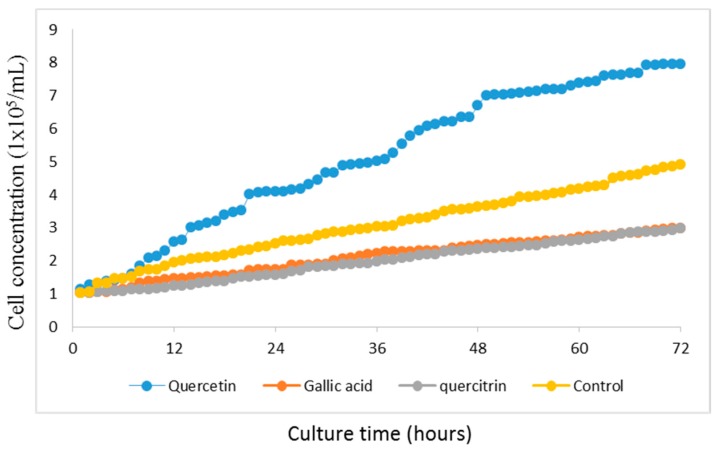
Real-time analysis of the effects of different fractions of PC on the growth of human gastric cancer cells GES-1. The whole culture period was three days.

The growth of human gastric cancer cells GES-1 is shown in Figure 3. Quercetin (10 μg/mL) promoted the cell growth and the cultured cells enter logarithmic growth phase after 24 h and grew at high speed after 72 h. In contrast, gallic acid (10 μg/mL) and quercitrin (10 μg/mL) showed inhibitory effects on cell growth. Quercetin was used in subsequent experiments.

### 2.3. Effects of Quercetin on GES-1 Cells

**Figure 4:**
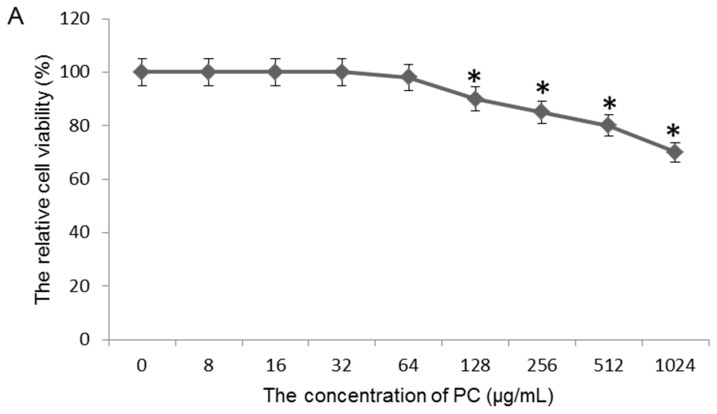

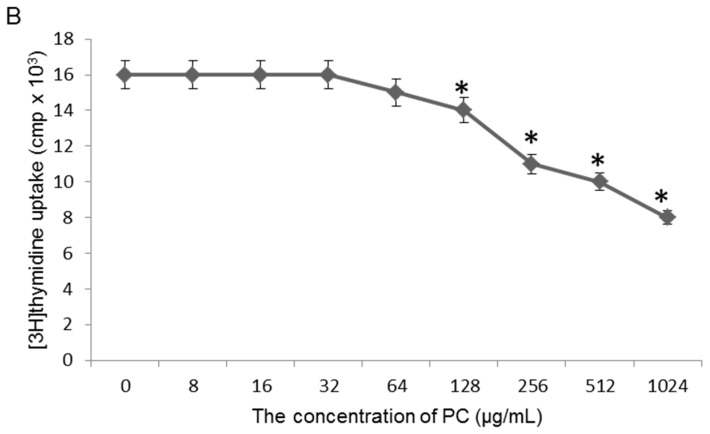
The effects of quercetin toxicity on cell viability of GES-1 cells after 48-h culture: (**A**) the cell viability of GES-1 cells was measured by trypan blue; and (**B**) the cell viability of GES cells was measured by [^3^H] thymidine incorporation analysis. The cell viability showed the results of five independent experiments, expressed as mean ± SD. * *p* < 0.05 vs. 0 μg/mL.

To avoid the toxicity of quercetin on GES-1 cells because of its high level, the concentration of quercetin was measured. With exposure of the GES-1 cells to 64 μg/mL quercetin, no loss of cell viability was observed after 48-h culture (*p* < 0.05) (Figure 4A). [^3^H] thymidine uptake is a simple way to measure cellular proliferation [28]. If cellular proliferation is stopped, the drug toxicity is often considered. The toxicity of quercetin was measured via measuring the [^3^H] thymidine in DNA after exposure to different concentration quercetin. As shown in Figure 4B, an obvious decrease in [^3^H] thymidine uptake was found when the concentration was more than 128 μg/mL (*p* < 0.05). Based on these results, we use 64 μg/mL of quercetin to treat models to avoid the cytotoxicity of high-level quercetin.

### 2.4. Assay of *H. pylori*-Infected Mouse Model

**Figure 5:**
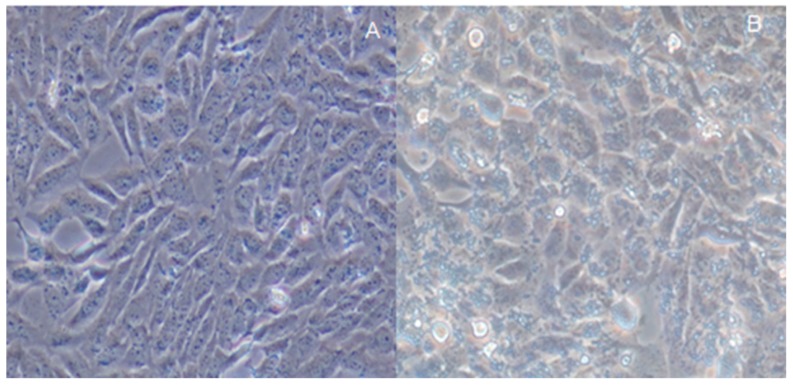
Infection assay of a mouse model: (**A**) gastric cells were cultured for 24 h and the cells were in a fine situation; and (**B**) gastric cells were destroyed after *H. pylori* infection.

There were significant morphological differences between gastric cells from healthy mice (Figure 5A) and those obtained from the mice infected with *H. pylori* (Figure 5B). Gastric cells were in a normal situation while their cell structures were destroyed after *H. pylori* infection. The levels of IL-8 increased 8.1-fold in a MG group when compared with a CG group (Table 1) (*p* < 0.05). All of the results showed that a mouse model was successfully established. In addition, quercetin treatment can reduce IL-8 level.

### 2.5. Effects of Quercetin on Mouse Model Inflammation

IL-8 was significantly higher in a MG group than in a QG group and reached a concentration of 1200.25 ± 16.43 pg/mL at 24 h (Table 1) (*p* < 0.05), and then gradually declined. After the addition of quercetin, IL-8 levels were lower in a QG group (655.12 ± 31.45 pg/mL) than in a MG group (*p* < 0.05). For uninfected mice, there was statistically significant difference when the concentrations of quercetin were more than 16 μg/mL (Table 2) (*p* < 0.05). 

### 2.6. The Effects of Quercetin on Cell Cycle and Apoptosis

**Figure 6:**
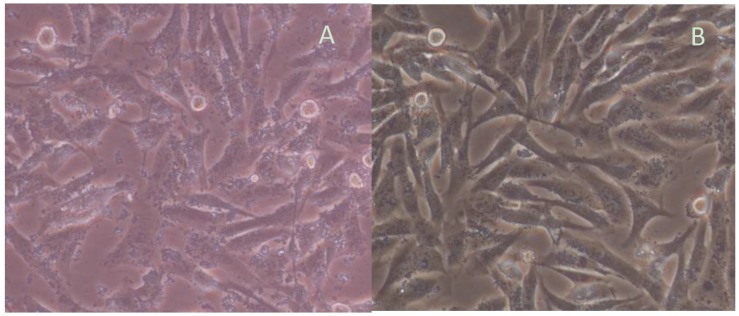
Assay of the gastric cells of mice model: (**A**) the cells from the mice infected with *H. pylori* were cultured for 24 h and the cells were destroyed; and (**B**) the effects of Quercetin on the cells from model mice.

The effect of *H. pylori* on gastric cell morphology of mice was observed under an inverted microscope. The results showed that normal cells were spindle, more regular, sharp edges, and adherent, and the growing state was good. After *H. pylori* infection, cell morphology changed into a larger size after 12-h co-culture, but the overall morphology of the cells remained intact with sharp edges. After 24 h, polymorphic cells appeared with obvious breaking granular forms (Figure 6).

**Figure 7:**
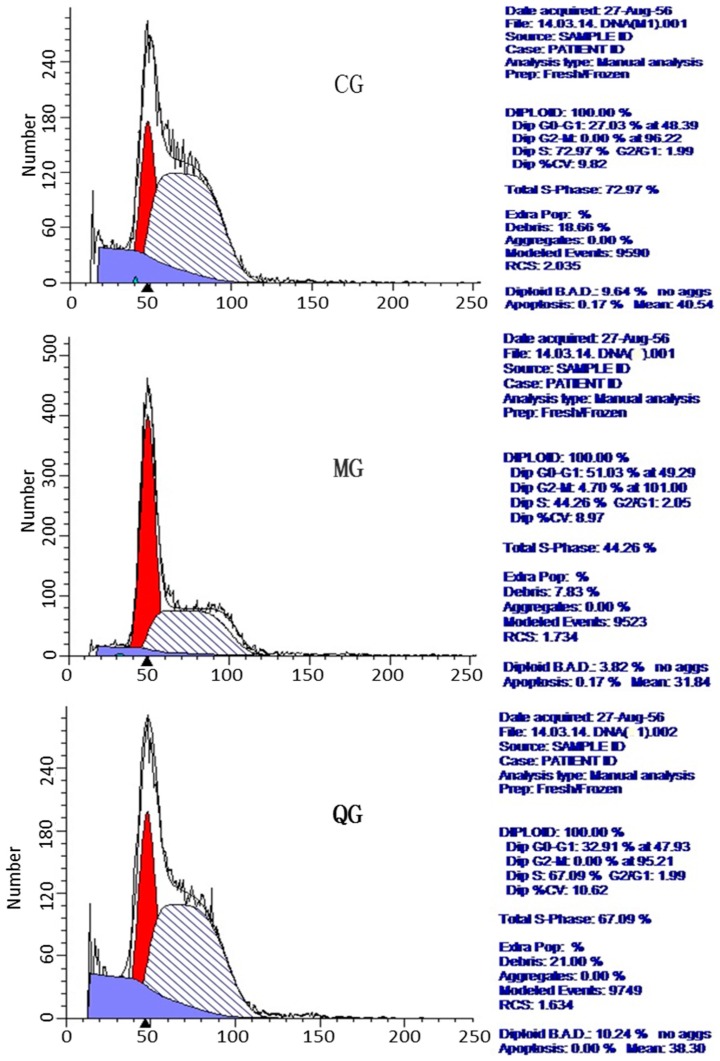
Effect of quercetin on the cell cycle of *H. pylori*-associated gastritis (HAG). CG, Diploid, 100%; Diploid G0–G1(phases of the cell cycle): 27.03% at 48.39; Diploid G2-M(phases of the cell cycle): 0.00% at 96.22; Diploid S: 72.97%; Diploid BAD (Background, Aggregates and Debris), 10.64%. No aggs (the number of aggregates); Apoptosis, 0.0%; Mean, 40.54; MG, Diploid, 100%; Diploid G0–G1: 51.03% at 49.29; Diploid G2-M: 4.70% AT 101.00; Diploid S: 44.26%; Diploid BAD, 3.87%; No aggs; Apoptosis, 5.24%; Mean, 31.84; QG, Diploid, 100%; Diploid G0–G1: 32.92% at 47.93; Diploid G2-M: 0.00% AT 95.21; Diploid S: 67.09%; Diploid BAD, 8.86%; No aggs; Apoptosis, 1.17%; Mean, 38.84.

FCM results showed that G0/G1 phase cells increased significantly and S-phase cells reduced in a MG group while G0/G1 phase cells reduced significantly and S-phase cells increased in a CG group (Figure 7, Table 3, *p* < 0.05). *H. pylori* can cause cell cycle arrest in G0/G1 phase while quercetin can make the cell cycle tend to be normally distributed to maintain normal cell proliferation (Figure 7, Table 3).

**Figure 8:**
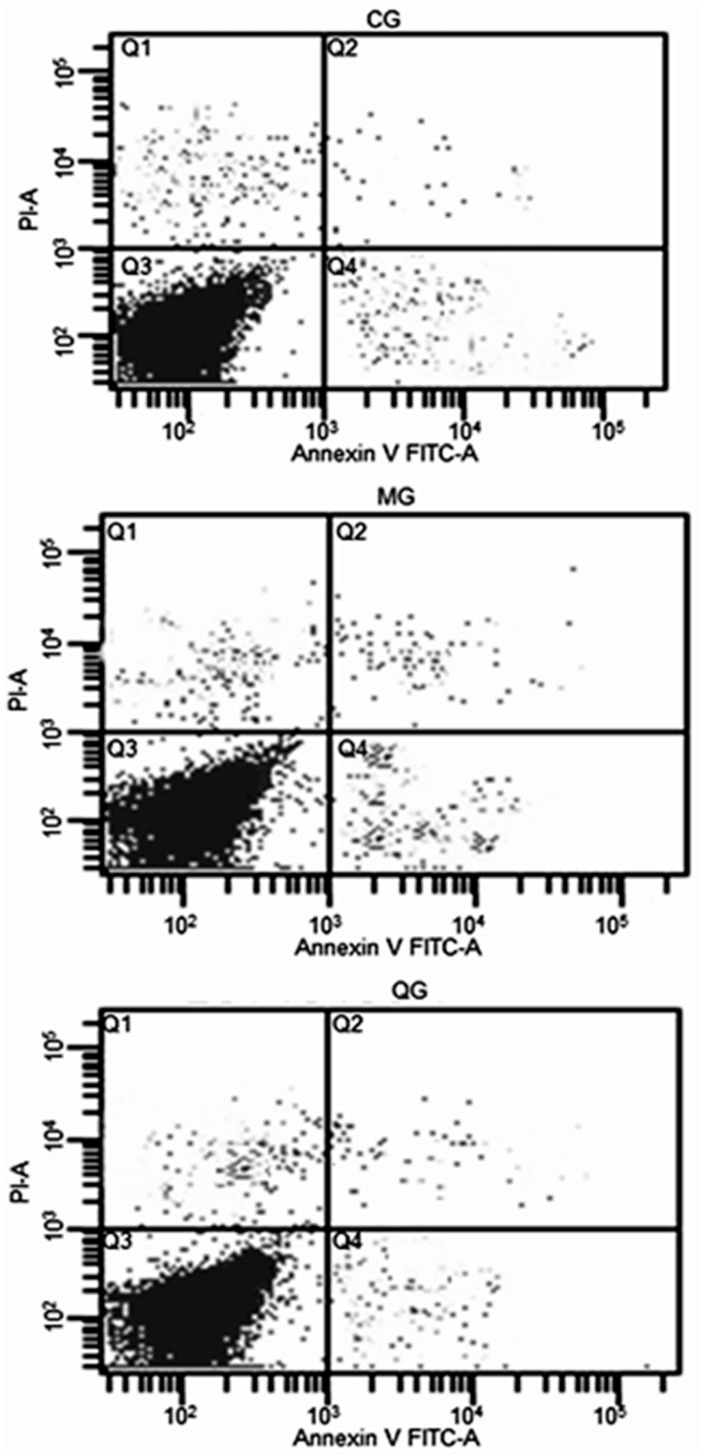
Effect of *Polygomun capitutam* on the cell apoptosis of HAG. CG, apoptosis, 0.2%; MG, apoptosis, 3.57%; QG, apoptosis, 1.2%.

FCM results showed that: apoptotic rate was the highest in a MG group while the rate was the lowest in a CG group (Figure 4). *H. pylori* increased GES-1 apoptosis while quercetin could inhibit the cell apoptosis caused by *H. pylori*. The apoptosis rate was lower in QG group than in a MG group (Figure 8).

### 2.7. The Effects of Quercetin on the mRNA Levels of p38MAPK, BCL-2 and BAX

**Figure 9:**
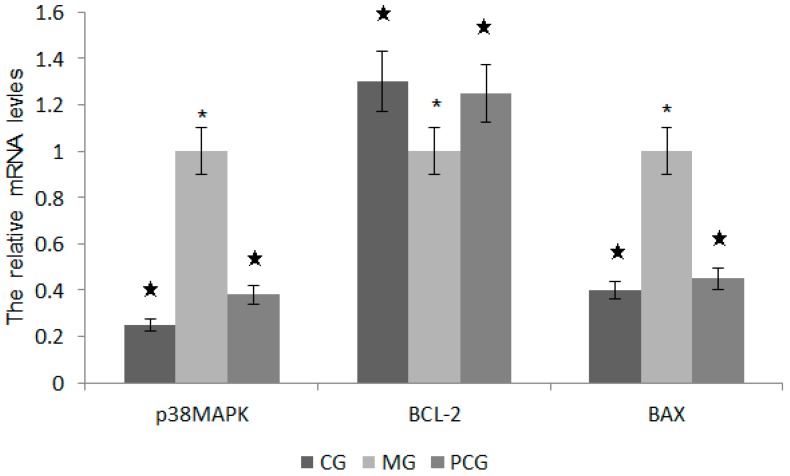
The relative mRNA levels of *p38MAPK*, *BCL-2* and *BAX. p38MAPK*, 38-kD tyrosine phosphorylated protein kinase; *BCL-2*, B cell lymphoma gene; *BAX*, apoptosis regulator BCL-2-associated protein X. * *p* < 0.05 vs. a CG group, *n* = 8. ^★^ *p* < 0.05 vs. a MG group, *n* = 8.

*C*t value and the initial amount of quantitative PCR melting curve were measured. Melting curve analysis was conducted for internal control gene β-action, the target genes p38MAPK, BCL-2, BAX, and all were specific to a single peak without nonspecific amplification products and primer dimers. Two-∆∆CT method was used to calculate the relative expression ratios. The levels of p38MAPK and BAX were lower in a QG group than in a MG Group (Figure 9, *p* < 0.05). In contrast, the level of BCL-2 significantly higher in a QG group than in a MG group (Figure 9, *p* > 0.05).

### 2.8. The Effects of Quercetin on the Protein Levels of BCL-2, BAX and Phosphated p38MAPK

**Figure 10:**
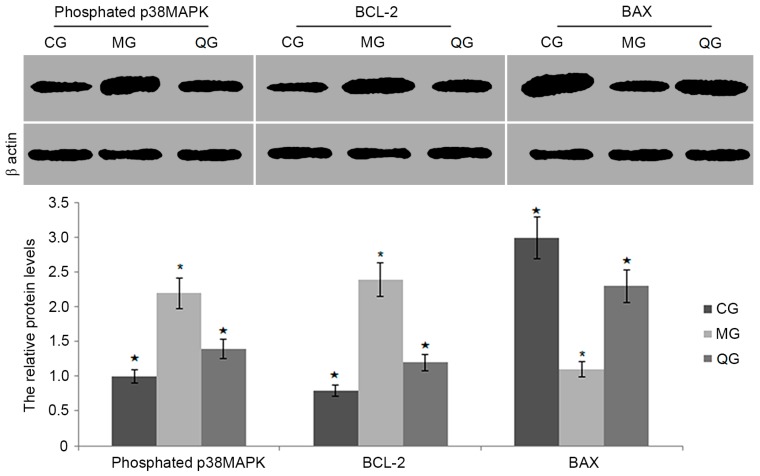
The relative protein levels of p38MAPK, BCL-2 and BAX. Note: * *p* < 0.05 vs. a CG group, *n* = 8. ^★^ *p* < 0.05 vs. a MG group, *n* = 8.

Quercetin treatment produced lower level of phosphorylated p38 MAPK (*p* < 0.05, Figure 10). Furthermore, quercetin reduced the protein level of BAX when compared with a model group (*p* < 0.05, Figure 10). In contrast, quercetin increased the protein level of BCL-2 when compared with a model group (*p* < 0.05, Figure 10).

The research for *H. pylori*-related digestive disease has become a hot spot since *H. pylori* can be found in the gastric tissues and successfully separated. *H. pylori* infection is an important gastroduodenal disease and responsible for the global burden of gastric cancer. However, with the *H. pylori* infection, clinical outcomes are not the same. Some infected persons develop into chronic gastritis, and some develop into more serious diseases such as stomach ulcers and stomach cancer. Proliferation balances gastric epithelial cell apoptosis and maintains the integrity of the gastric mucosa, while *H. pylori* infection can destroy this balance and lead to various gastric disorders. When the apoptosis is dominant, acute ulcers and or chronic atrophic gastritis will be induced. In contrast, gastric cancer will be induced when cell proliferation is dominant [29].

Western medicine has good effects on *H. pylori* infection, but the high incidence of side effects (5–20%), poor compliance, relapse rate, and long-term use of antibiotics can lead to intestinal flora disturbance. Furthermore, with the widespread use of medical therapy for *H. pylori*, drug-resistant *H. pylori* have been increased [30,31]. Chinese medicine has been used to eradicate *H. pylori* infection and show good effects with little unwanted adverse [32].

In this study, the biological characteristics of infected and control mice were analyzed to explore the cell’s regulatory mechanism for *H. pylori* infection. The levels of IL-8 were higher in a MG group than in a QG group and a CG group. The results suggest that *H. pylori* infection induces inflammatory response in gastric mucosal cells. *H. pylori*-infected gastric epithelial cells supplied a useful model in vitro for exploring biological characteristics after *H. pylori* infection. Furthermore, most gastric adenocarcinoma cell lines, such as AGS, MKN-45, HGC-27, MCG-823 and SGC-7901 [33], have been used to evaluate *H. pylori*-infected models to study the interaction between in *H. pylori* and gastric epithelial cells. However, an infected model seems to be better than these cells. 

Cell cycle is the fundamental process of cell activity. Quiescent cell cycle includes early DNA/DNA synthesis phase (G0/G1), DNA synthesis phase (S), late DNA synthesis phase (G2) and mitosis phase (M). Cell cycle and apoptosis is an important way to maintain the balance between cell proliferation and death. The growth of gastric epithelial cells is regulated by the cell cycle progression. Present FCM test results showed that G0/G1 phase cells significantly increased and S phase decreased in a model group when compared with a CG group, which suggested that *H. pylori* can induce cell cycle arrest in G0/G1. The results are somewhat surprising since the cells accumulated only in G0/G1. According to a previous report, influenza A virus replication can induce cell cycle arrest in G0/G1 phase [34]. Other research shows that *H. pylori* infection inhibits the level of miR-372 and miR-373 synthesis, resulting in the release of large tumor suppressor homolog 2, which causes a cell cycle arrest at the G1/S transition [35]. In a QG group, G0/G1 phase reduced and S phase increased when compared with a MG group, which indicated that quercetin could promote cell conversion from G0/G1 to S phase in a cell cycle and reduced the inhibition caused *H. pylori*. Therefore, quercetin can enhance the proliferation of gastric mucosal cells by promoting cell cycle progression.

Maintaining the basic structure of the gastric mucosa is dependent on cell loss and regeneration. Normal regeneration of gastric epithelial cell is generally 3–5 days. In acute gastric mucosal injury diseases, the imbalance cell loss and regeneration is mainly caused by the sharp increase via apoptosis in cell loss. Present studies confirmed that *H. pylori* caused serious damage to structure and function of gastric cells, which was mainly characterized by an increase of apoptosis in cell. The apoptosis can destroy the gastric mucosal barrier function, which is closely related with the structure in gastric tissue [36].

The occurrence of apoptosis can be caused by many factors and the process is also more complicated. Previous work found that peptic ulcer diseases and gastric ulcers are caused by the gastric epithelial cell apoptosis after *H. pylori* infection, which delays the process of healing and regeneration of gastric cells [37,38]. Present experiment initially confirms that quercetin has certain anti-apoptotic effects on gastric mucosal cells. The results showed that apoptosis rate of *H. pylori* infection was significantly higher in a MG group than in a QG group, suggesting that quercetin can significantly reduce apoptosis (Figure 4). Quercetin can be served to reduce the incidence of *H. pylori* infection by reducing the incidence of cell apoptosis.

The regulation of apoptosis involves in a variety of genes. In *H. pylori*-infected cells, the levels of p38MAPK and BAX were significantly higher in a MG group than in a QG group. p38MAPK as a kind of serine/threonine protein kinases, is distributed in the cytoplasm with a dual phosphorylation of serine and tyrosine protein kinase capacity. p38MAPK signaling pathway involves in a variety of physiological and pathological processes, such as mediating cell growth, development, division and differentiation. The present study showed that *H. pylori* infection could lead to the apoptosis of gastric epithelial cells by activating p38MAPK pathway (Figure 6). BCL-2 and BAX belong to the mitochondrial apoptotic pathways, which are mediated by BCL-2 gene family members. BAX and BCL-2 usually can form homodimers or heterodimers (BAX · BAX, BAX · BCL-2 and BCL-2 · BCL-2). Previous research shows that BAX.BCL-2 heterodimer formation is an important part during apoptosis process. The increasing ratio of BCL-2 and BAX inhibits the release of mitochondrial cytochrome C and caspase cascade, and ultimately inhibits apoptosis [39]. Present findings showed that the levels of BCL-2 were significantly higher in a QG group than in a MG group while the levels of BAX were significantly lower in a QG group than in a MG group (Figure 5 and Figure 6). The changes resulted in the increased of BCL-2/BAX ratio and inhibition of apoptosis. Therefore, quercetin may reduce apoptosis by down-regulating the levels of p38MAPK and BAX, decrease cell susceptibility to apoptosis signal, and ultimately suppress the apoptosis of a mouse model.

## 3. Materials and Methods

### 3.1. Materials 

*H. pylori* strain SS2000 was kindly donated by Professor Jianzhong Zhang from Chinese Center for Disease Control and Prevention (Beijing, China). Human-derived immortalized gastric epithelial cells (GES-1), was purchased from Beijing Institute for Cancer Research (Beijing, China). PC extract was prepared according to a previous report [40]. Dried PC (5 g) were extracted in 50 mL 50% ethanol at 25 °C for half an hour. The extract from supernatant was collected after centrifuge at 15,000 rpm for five min. The supernatant was lyophilized concentrated by using a RE52-3 rotary evaporator (Shanghai Huxi Instrumental Company, Shanghai, China). Fetal bovine serum (FBS) and Dulbecco’s High Glucose Modified Eagles Medium (DMEM) was purchased from Hyclone (Hyclone, Logan, CA, USA). TRIzol was from Invitrogen Company (Invitrogen, Carlsbad, CA, USA). TaKaRa RNA reverse transcription and Quantitative PCR Kits were from Takara Biotechnology (Dalian, China) Co., Ltd. (Dalian, China). KGI staining apoptosis detection kit was from Nanjing KGI biotechnology Co., Ltd. (Nanjing, China).

### 3.2. Polyphenol and Ascorbic Acid Extracts

The raw extracts of PC were subjected to the extraction with 20-mL 20% ethanol for 4 h. The mixture was filtrated with a membrane NMWL of 3 kDa (Millipore, Billerica, MA, USA). The filtrated liquid was dried and dissolved in 20-μL methanol.

### 3.3. Bioactive Component Isolation by Preparative HPLC

A ten-μL above aliquot was injected into a reversed-phase HPLC (Waters, Milford, MA, USA). The column was eluted by 30% ethanol and linearly increased to 100% at a flow rate of one mL/min. The peaks were detected at 360 nm. According to the peak time, each individual identifiable peak was collected via a detector. The collected fraction was dried and resolved in 20 μL of ethanol. 

### 3.4. Mass Spectrometry

Each fraction was analyzed by Micromass ESI Mass Spectrometer (Waters). Source temperature was set at 100 °C, and the desolation gas temperature was set at 350 °C. Nitrogen (99.99% purity) and argon (99.99% purity). Desolation gas flow was set at 650 L/h and cone gas flow was set at 50 L/h, respectively. The sampling cone was set to 25 V and the capillary voltage was set to 3 kV. Sodium format was used to calibrate mass spectrometer in both positive- and negative-ion modes. The mass spectrometer was set over the range *m*/*z* 0–500.

### 3.5. H. pylori Seed Culture

*H. pylori* SS2000 was inoculated on agar plates with a sterile pipette containing 10% sterile defibrinated sheep blood, at 37 °C micro-aerobic conditions (5% O_2_, 10% CO_2_, 85% N_2_, 90% of humidity). After 72 h, *H. pylori* was confirmed with visual observation for bacterial morphology, fast urease assays and micro-aerobic tests. With freshly prepared bacterial suspension in PBS, bacterial concentration was measured on a micro-plate reader (the amount of bacteria = 2.2 × 10^8^ × OD value × dilution factor), and diluted to a final concentration of 3 × 10^8^ CFU/mL.

### 3.6. Subculture of GES-1 Cells

Frozen human gastric cancer cells GES-1 were removed quickly from −80 °C liquid nitrogen bottle and placed in 37 °C water bath. After appropriate dilution with medium, the cells were inoculated in flasks. When the cells covered more than 80% of the flask surface, the culture medium was removed, and rinsed with PBS after 0.25% trypsin digestion. Cells were observed under a microscope, and medium was added repeatedly until all of the cells were in a suspension. The cell pellets were diluted into 1 × 10^7^/mL aliquots to several cell culture flasks and cultured.

GES-1 cells were seeded in 96-well plates with each well of 1.0 × 10^4^ cells. Real-time cell growth was measured by xCELLigence (Roche, IN, USA). The whole period was 72 h and growth curve was setup according the cell concentrations.

### 3.7. Toxicity Test

The toxicity of quercetin was tested via the viability of GES-1 cells. The viability of GES-1 cells was measured by trypan blue (Cat. No., T0556, TCI (Shanghai, China) Development Co., Ltd., Shanghai, China) uptake and [^3^H]thymidine (Beijing Atomic Energy Research Institute, Beijing, China) incorporation after the cells were treated with different concentrations of quercetin. A total 200 mg extract powder was dissolved in one-mL ethanol, and serially diluted into 80 μg/mL, 160 μg/mL, 320 μg/mL, 640 μg/mL, 1280 μg/mL, 2560 μg/mL, 5120 μg/mL and 10240 μg/mL with water. Meanwhile, the ethanol concentration was adjusted to 5%. Different concentrations of quercetin with equal volume were added to different wells with a final concentration 8 μg/mL, 16 μg/mL, 32 μg/mL, 64 μg/mL, 128 μg/mL, 256 μg/mL, 512 μg/mL, and 1024 μg/mL. The viable cells were counted using Trypan Blue. For [^3^H]thymidine uptake assay, 200 μL of a cell suspension of 1.0 × 10^5^ cells/mL were added to each well of a 96-well plate. The concentration of cells was calculated when exposed to different concentrations of quercetin. After-24 h culture, cells were treated with one Ci/well of [^3^H]thymidine (67 Ci/mmol) for 4 h, and then collected with an automated harvester (Mash II Model, Microbiological Associates, Rockville, MD, USA), and measured by a liquid-scintillation analyzer (XH27-FJ-2107P, MIDWEST-G, Beijing, China).

### 3.8. H. pylori-Infected Mouse Model

Eighteen male Kunming mice (4-week old, 20–25 g) were purchased from animal center of Guizhou medical university (Guiyang, China). All mice were maintained under 12 h light/12 h dark at 21–24 °C with relative 65% humidity. The mice were fed with the diet shown in Table 4. *H. pylori* was prepared as suspension using high glucose DMEM and diluted to obtain 3 × 10^8^ CFU/mL using a 6-cell plate. The diets were mixed with *H. pylori* at 1 mL/kg. The mice were fed with the mixture diets for one day and kept at the same condition. After 3 days, two models and two healthy ones were killed by decapitation. The majority of *H. pylori*-infected mice developed duodenitis and necrotic nematodes in small intestine but no symptoms in healthy ones.

### 3.9. Identification of H. pylori 

In a urease test, the urease reagent was dropped on a sterile glass slide with a sterile loop of *H. pylori* was taken and mixed with the reagent. The orange-yellow would be changed into red rose within 2 min if urease test was positive. The *H. pylori* was plated on a Columbia blood agar plates with sterile L-type glass rod and cultured for 3 days. The *H. pylori* strains were identified from the feces of the mice.

### 3.10. Grouping 

Eight healthy mice and 16 *H. pylori*-infected mice were random assigned into three groups: healthy group (CG), model group (MG), and quercetin treated group (QG, the model mice were fed with 64 mg/kg quercetin for 72 h). Five hundred microliters of blood was obtained from the tail, serum was isolated and the expression of IL-8 was measured by enzyme-linked immunosorbent assay (ELISA).

### 3.11. Flow Cytometry Detection of Cell Cycle and Apoptosis

After three weeks, all mice were killed by decapitation and small gastric tissues were obtained at the same place. Single cells were prepared according to a previous report [41]. The cells were washed twice with PBS, centrifuged at 2000 rpm for 5 min and adjusted to the cell concentration 1 × 10^5^/mL; The supernatant was removed, 70% of the volume of cold ethanol was used for 2 h–12 h, 4 °C preservation, and washed with PBS before staining. One hundred microliters of RNaseA was added and cultured at 37 °C water for 30 min. Four hundred microliters of PI was added and culture in dark at 4 °C for 30 min. FACSCalibur flow cytometer was used to detect cell cycle distribution. The cells were washed twice with PBS and centrifuged at 2000 rpm for 5 min). Five hundred microliters of binding buffer was used to prepare cell suspension at 1 × 10^5^ cells/mL. Fifty microliters of Annexin V-FITC was added, mixed, and 5 μL propidium iodide was added and inoculated at room temperature for 5–15 min; FACSCalibur flow cytometer was used to detect cell apoptosis.

### 3.12. Real Time-PCR Assay for p38MAPK, BCL-2 and BAX

The total RNA was isolated by using a RNA purification kit according to manufacturer’s instruction. The purity and concentration of RNA was detected using UV spectrophotometer. The cDNAs were synthesized from purified RNA by using a Reverse Transcription Kit. The mRNA levels of p38MAPK, BCL-2, BAX were measured by using the primers synthesized as follows: p38MAPK, sense primer, 5′-TCCAGACCATTTCAGTCCATC-3′ and anti-sense primer, 5′-CGTCCAACAGACCAATCACA-3′, 100 bp; BAX, sense primer, 5′-GTGCACCAAGGTGCCGGAAC-3′ and anti-sense primer, 5′-TCAGCCCATCTTCTTCCAGA-3′, 111 bp; BCL-2, sense primer, 5′-ATGTGTGTGGAGAGCGTCAA-3′ and anti-sense primer, 5′-AGAGACAGCCAGGAGAAATCA-3′, 182 bp; β-actin, sense primer, 5′-CAGGGCGTGATGGTGGGCATG-3′ and anti-sense primer, 5′-GCCACACGCAGCTCATTGTA-3′, 170 bp. For quantitative reverse transcription PCR (qRT-PCR), β-actin was used as the normalizer, and cells from uninfected GES-1 were served as blank controls. All cDNA samples were analyzed in triplicate, along with β-actin controls. Levels of mRNA were compared between *H. pylori*-infected and uninfected GES-1. Relative units were calculated as 2-^∆∆^*C*t (*C*t, cycle threshold) where ^∆∆^*C*t is equal to the difference between the ^∆^*C*t of the gene of interest of the experimental sample and the calibrator tissue. The ^∆^*C*t of target genes was calculated as the difference between the cycle threshold of target genes and the cycle threshold of β-actin. 

PCR amplification was performed with an initial denaturation cycle at 95 °C for 5 min, followed by 50 amplification cycles consisting of 95 °C for 5 s, annealing at 60 °C for 10 s, and extension at 72 °C for 20 s. After amplification, a melting step was performed, consisting of 95 °C for 5 s, cooling to 45 °C for 30 s (with a temperature transition rate of 20 °C per second), and finally a slow rise in the temperature to 85 °C at a rate of 0.1 °C per second with a fluorescence decline.

### 3.13. Western Blot

Cells were lysed in lysis buffer containing 20 mM Tris-HCl (pH 8.0), 100 mM NaCl, 0.1% Triton X-100, 10 mM EDTA, 0.1% Sodium dodecyl sulfate (SDS, Cat. No., L4509, Sigma-Aldrich, St. Louis, MO, USA), 50 mM sodium fluoride (NaF, Cat. No., S7920, Sigma-Aldrich, St. Louis, MO, USA), 100 μM phosphatase inhibitor Sodium orthovanadate (Cat. No., S6508, Sigma-Aldrich), and 100 μM phenylmethylsulfonyl fluoride (PMSF, Cat. No., P7626, Sigma-Aldrich) and cellular proteins were subjected to SDS-PAGE, transferred to PVDF membranes (Cat. No. RPN303F, Millipore), blocked by 5% nonfat dried milk in TBS buffer (20 mM Tris-HCl and 0.1 M NaCl, pH 8.0), and incubated with the antibodies [rabbit anti-human phospho-p38 MAPK (Thr180/Tyr182) polyclonal antibody, 1:2000 dilutions, Cat. No., 9211, CST (Shanghai, China) Biological Reagents, Shanghai, China; Rabbit anti-human Bax polyclonal antibody at 1/3000 dilution, Cat. No., ab10813, Abcam, Cambridge, MA, USA; Rabbit anti-human BCL-2 monoclonal antibody at 1/3000 dilution, Cat. No., ab32124, Abcam, Cambridge, MA, USA; Rabbit anti-human beta actin polyclonal antibody at 1/3000 dilution, Cat. No., ab189073, Abcam, Cambridge, MA, USA; HRP labelled goat anti-rabbit second antibody at 1/2000 dilution, Cat. No., ab97051, Abcam, Cambridge, MA, USA] in the TBST buffer containing 0.1% Tween 20, one percent nonfat dried milk, 20 mM Tris-HCl and 0.1 M NaCl, pH8.0). Immunoreactive bands were visualized by the Enhanced Chemiluminescence Plus (Amersham ECL plus, GE Healthcare, Piscataway, NJ, USA).

### 3.14. Statistical Analysis

All data were analyzed via SPSS 20.0 (Chicago, IL, USA). Histograms and the Kolmogorov–Smirnov methods were conducted to determine a normal distribution of the variables. With a normal distribution, quantitative data were presented as mean ± SD. *t*-test for independent means is used to test whether there is a difference between groups. *p* < 0.05 was regarded as statistically significant.

## 4. Conclusions

Quercetin shows no cytotoxicity to GES-1 when its concentration is less than 64 μg/mL. Quercetin regulates the balance of cell proliferation and apoptosis to protect the gastric epithelia. Quercetin can control apoptosis rate of gastric cells by regulating the levels of p38MAPK, BCL-2 and BAX genes. Thus, quercetin could be a new drug candidate for treating *H. pylori* infection related diseases.

## Figures and Tables

**Table 1:** Effect of *H. pylori* on the secretion of interleukin-8 (IL-8).

Groups	24 h
CG	150.33 ± 43.13 *
MG	1200.25 ± 16.43 ^★^
QG	655.12 ± 31.45 ^★,^*

Note: The concentration of quercetin was 64 μg/mL in a quercetin treated group. ^★^ *p* < 0.05 vs. a control group and * *p* < 0.05 vs. a cell model group, *n* = 8.

**Table 2:** The effect of *H. pylori* on IL-8 production (pg/mL).

Groups	Concentration of Quercetin	24 h	48 h	72 h
*H. pylori*-infected mice	0 μg/mL	1200.25 ± 16.43	1153.26 ± 30.25	1103.14 ± 21.32
	8 μg/mL	1099.42 ± 21.34 *	1047.08 ± 38.19 *	990.13 ± 19.63 *
	16 μg/mL	959.47 ± 23.16 *	900.92 ± 26.02 *	860.08 ± 21.36 *
	32 μg/mL	830.36 ± 32.17 *	787.92 ± 27.32 *	743.25 ± 12.50 *
	64 μg/mL	760.34 ± 21.65 *	656.58 ± 13.96 *	590.63 ± 19.75 *
Uninfected mice	0 μg/mL	150.33 ± 43.13	142.39 ± 37.44	160.26 ± 30.18
	8 μg/mL	149.32 ± 32.17	152.32 ± 39.76	140.64 ± 31.73
	16 μg/mL	139.56 ± 21.87 *	134.72 ± 25.98 *	136.45 ± 23.76 *
	32 μg/mL	130.12 ± 21.35 *	138.43 ± 21.79	132.46 ± 18.73 *
	64 μg/mL	121.17 ± 15.88 *	126.34 ± 16.24 *	128.42 ± 18.37 *

Note: * *p* < 0.05 vs. a group without quercetin treatment, *n* = 8.

**Table 3:** Effects of *Polygomun capitutam* on the cell cycle.

Groups	DipG0–G1%	DipG2-M%	DipS%
CG	29.48 ± 2.18 *	0 *	70.52 ± 3.31 *
MG	50.35 ± 1.38 ^★^	4.82 ± 0.40 ^★^	44.83 ± 3.02 ^★^
PCG	31.07 ± 1.60 *	2.14 ± 0.72 *	66.79 ± 0.38 *

Note: * *p* < 0.05 vs. a MG group, *n* = 6. ^★^ *p* < 0.05 vs. a CG group, *n* = 6.

**Table 4:** Ingredients of the diets consumed by mice.

wt %	
Casein	23.1 ± 2.1
Corn starch	43.9 ± 4.1
Sucrose	5.1 ± 0.5
Maltodextrin	5.3 ± 0.5
Soy oil	5.1 ± 0.4
Palm oil	–
Lard	–
Cellulose	5.1 ± 0.6
Mineral mixture	6.8 ± 0.7
Vitamin mixture	1.3 ± 0.1
Kj %	
Protein	23.2 ± 2.3
Fat	12.5 ± 1.2
Carbohydrates	65.3 ± 3.4
Energy content [kJ/g]	15.3 ± 1.6
**Mean sterol contents, mg/100 g**	
Cholesterol	0.52 ± 0.08
Campesterol	1.21 ± 0.16
Stigmasterol	2.13 ± 0.31
Sitosterol	3.04 ± 0.52
∆^5^-Avenasterol	0.18±0.02
Sitostanol	1.30 ± 0.32
24-Methylene cycloartenol	0.31 ± 0.04
Cycloartenol	Not detectable
Campestanol	0.41 ± 0.10
Total sterols	8.50 ± 0.79

Note: The batches were performed in five separate blocks.

## References

[1] Nguyen T.T., Kim S.J., Park J.M., Hahm K.B., Lee H.J. (2015). Repressed TGF-beta signaling through CagA-Smad3 interaction as pathogenic mechanisms of *Helicobacter pylori*-associated gastritis. J. Clin. Biochem. Nutr..

[2] Sonnenberg A., Genta R.M. (2016). Inverse Association between *Helicobacter pylori* Gastritis and Microscopic Colitis. Inflamm. Bowel. Dis..

[3] Wang Z.H., Gao Q.Y., Fang J.Y. (2013). Meta-analysis of the efficacy and safety of Lactobacillus-containing and Bifidobacterium-containing probiotic compound preparation in *Helicobacter pylori* eradication therapy. J. Clin. Gastroenterol..

[4] Omata F., Shimbo T., Ohde S., Deshpande G.A., Fukui T. (2017). Cost-Effectiveness Analysis of *Helicobacter pylori* Diagnostic Methods in Patients with Atrophic Gastritis. Gastroenterol. Res. Pract..

[5] Cheng H.C., Tsai Y.C., Yang H.B., Yeh Y.C., Chang W.L., Kuo H.Y., Lu C.C., Sheu B.S. (2017). The corpus-predominant gastritis index can be an early and reversible marker to identify the gastric cancer risk of *Helicobacter pylori*-infected nonulcer dyspepsia. Helicobacter.

[6] Chmiela M., Karwowska Z., Gonciarz W., Allushi B., Staczek P. (2017). Host pathogen interactions in *Helicobacter pylori* related gastric cancer. World J. Gastroenterol..

[7] Ishaq S., Nunn L. (2015). *Helicobacter pylori* and gastric cancer: A state of the art review. Gastroenterol. Hepatol. Bed Bench.

[8] Burkitt M.D., Duckworth C.A., Williams J.M., Pritchard D.M. (2017). *Helicobacter pylori*-induced gastric pathology: Insights from in vivo and ex vivo models. Dis. Model. Mech..

[9] Moriya K., Tamura H., Nakamura K., Hosone M., Inokuchi K. (2017). A primary esophageal MALT lymphoma patient with *Helicobacter pylori* infection achieved complete remission after *H. pylori* eradication without anti-lymphoma treatment. Leuk. Res. Rep..

[10] Cekin A.H., Sahinturk Y., Akbay Harmandar F., Uyar S., Yolcular B.O., Cekin Y. (2017). Use of probiotics as an adjuvant to sequential *H. pylori* eradication therapy: Impact on eradication rates, treatment resistance, treatment-related side effects, and patient compliance. Turk. J. Gastroenterol..

[11] Kwon S.B., Lee K.L., Kim J.S., Lee J.K., Kim W., Jung Y.J., Jeong J.B., Kim J.W., Kim B.G. (2010). Antibiotics-associated diarrhea and other gastrointestinal abnormal responses regarding *Helicobacter pylori* eradication. Korean J. Gastroenterol..

[12] Tamayo E., Montes M., Fernandez-Reyes M., Lizasoain J., Ibarra B., Mendarte U., Zapata E., Mendiola J., Perez-Trallero E. (2017). Clarithromycin resistance in *Helicobacter pylori* and its molecular determinants in northern Spain, 2013–2015. J. Glob. Antimicrob. Resist..

[13] Boehnke K.F., Valdivieso M., Bussalleu A., Sexton R., Thompson K.C., Osorio S., Reyes I.N., Crowley J.J., Baker L.H., Xi C. (2017). Antibiotic resistance among *Helicobacter pylori* clinical isolates in Lima, Peru. Infect. Drug Resist..

[14] Meng F., Yang S., Wang X., Chen T., Wang X., Tang X., Zhang R., Shen L. (2017). Reclamation of Chinese herb residues using probiotics and evaluation of their beneficial effect on pathogen infection. J. Infect. Public Health.

[15] Si L., Li P., Liu X., Luo L. (2016). Chinese herb medicine against Sortase A catalyzed transformations, a key role in gram-positive bacterial infection progress. J. Enzym. Inhib. Med. Chem..

[16] Higuchi K., Watanabe T., Tanigawa T., Tominaga K., Fujiwara Y., Arakawa T. (2010). Sofalcone, a gastroprotective drug, promotes gastric ulcer healing following eradication therapy for *Helicobacter pylori*: A randomized controlled comparative trial with cimetidine, an H2-receptor antagonist. J. Gastroenterol. Hepatol..

[17] Higuchi K., Tanigawa T., Hamaguchi M., Takashima T., Sasaki E., Shiba M., Tominaga K., Fujiwara Y., Oshitani N., Matsumoto T. (2003). Comparison of the effects of rebamipide with those of cimetidine on chronic gastritis associated with *Helicobacter pylori* in Mongolian gerbils. Aliment. Pharmacol. Ther..

[18] Previti F.W., Holt R.W. (1980). Failure of cimetidine prophylaxis in the critically ill patient: Two fatal cases of duodenal perforation. J. Med. Soc. N. J..

[19] Zhang K., Zhang J., Wei S., Jing W., Wang Y., Liu A. (2013). Development and validation of HPLC coupled with triple quadrupole MS for the simultaneous determination of six phenolic acids, six flavonoids, and a lignan in *Polygonum capitatum*. J. Sep. Sci..

[20] Li X., Yu M., Meng D., Li Z., Zhang L. (2007). A new chromone glycoside from *Polygonum capitatum*. Fitoterapia.

[21] Liao S.G., Zhang L.J., Sun F., Zhang J.J., Chen A.Y., Lan Y.Y., Li Y.J., Wang A.M., He X., Xiong Y. (2011). Antibacterial and anti-inflammatory effects of extracts and fractions from *Polygonum capitatum*. J. Ethnopharmacol..

[22] Dong X., Fu J., Yin X., Li X., Wang B., Cao S., Zhang J., Zhang H., Zhao Y., Ni J. (2014). Pharmacological and other Bioactivities of the Genus Polygonum—A Review. Trop. J. Pharm. Res..

[23] Liu Z.J., Qi J., Zhu D.N., Yu B.Y. (2008). Chemical constituents from *Polygonum capitatum* and their antioxidation activities in vitro. Zhong Yao Cai.

[24] Wang H., Sun Y., Liu S., Yu H., Li W., Zeng J., Chen C., Jia J. (2011). Upregulation of progranulin by *Helicobacter pylori* in human gastric epithelial cells via p38MAPK and MEK1/2 signaling pathway: Role in epithelial cell proliferation and migration. FEMS Immunol. Med. Microbiol..

[25] Ryszczuk E., Roszko-Kirpsza I., Guzinska-Ustymowicz K., Olejnik B.J., Kaczmarski M.G., Maciorkowska E. (2016). EGFR and Bcl-2 in gastric mucosa of children infected with *Helicobacter pylori*. Postepy Hig. Med. Dosw..

[26] Bartchewsky W., Martini M.R., Squassoni A.C., Alvarez M.C., Ladeira M.S., Salvatore D.M., Trevisan M.A., Pedrazzoli J., Ribeiro M.L. (2010). Effects of *Helicobacter pylori* infection on the expressions of Bax and Bcl-2 in patients with chronic gastritis and gastric cancer. Dig. Dis. Sci..

[27] Huang Y., Sun H.-Y., Qin X.-L., Li Y.-J., Liao S.-G., Gong Z.-P., Lu Y., Wang Y.-L., Wang A.-M., Lan Y.-Y. (2017). A UPLC-MS/MS Method for Simultaneous Determination of Free and Total Forms of a Phenolic Acid and Two Flavonoids in Rat Plasma and Its Application to Comparative Pharmacokinetic Studies of *Polygonum capitatum* Extract in Rats. Molecules.

[28] Ahmed S.A., Gogal R.M., Walsh J.E. (1994). A new rapid and simple non-radioactive assay to monitor and determine the proliferation of lymphocytes: An alternative to [^3^H] thymidine incorporation assay. J. Immunol. Methods.

[29] Naumann M., Crabtree J.E. (2004). *Helicobacter pylori*-induced epithelial cell signalling in gastric carcinogenesis. Trends. Microbiol..

[30] Boyanova L., Evstatiev I., Gergova G., Yaneva P., Mitov I. (2015). Linezolid susceptibility in *Helicobacter pylori*, including strains with multidrug resistance. Int. J. Antimicrob. Agents.

[31] Ghotaslou R., Leylabadlo H.E., Asl Y.M. (2015). Prevalence of antibiotic resistance in *Helicobacter pylori*: A recent literature review. World J. Methodol..

[32] Xu L.W., Jia M., Salchow R., Kentsch M., Cui X.J., Deng H.Y., Sun Z.J., Kluwe L. (2012). Efficacy and side effects of chinese herbal medicine for menopausal symptoms: A critical review. Evid. Based Complement. Altern. Med..

[33] Ran Z.H., Liu J., Feng Y., Zou J., Xiao S.D. (2004). Investigation of the sensitivities of distinct gastric cancer cells to parvovirus H-1 induced cytotoxicity. Chin. J. Dig. Dis..

[34] He Y., Xu K., Keiner B., Zhou J., Czudai V., Li T., Chen Z., Liu J., Klenk H.D., Shu Y.L., Sun B. (2010). Influenza A virus replication induces cell cycle arrest in G0/G1 phase. J. Virol..

[35] Belair C., Baud J., Chabas S., Sharma C.M., Vogel J., Staedel C., Darfeuille F. (2011). *Helicobacter pylori* interferes with an embryonic stem cell micro RNA cluster to block cell cycle progression. Silence.

[36] Tanaka S., Saito K., Reed J.C. (1993). Structure-function analysis of the Bcl-2 oncoprotein. Addition of a heterologous transmembrane domain to portions of the Bcl-2 beta protein restores function as a regulator of cell survival. J. Biol. Chem..

[37] Chang H., Chen D., Ni B., Zuo Q., Wang C., Han R., Lan C. (2016). Cortactin Mediates Apoptosis of Gastric Epithelial Cells Induced by VacA Protein of *Helicobacter pylori*. Dig. Dis. Sci..

[38] Zhao H., Zhu H., Lin Z., Lin G., Lv G. (2015). Compound 13, an alpha1-selective small molecule activator of AMPK, inhibits *Helicobacter pylori*-induced oxidative stresses and gastric epithelial cell apoptosis. Biochem. Biophys. Res. Commun..

[39] Leri A., Claudio P.P., Li Q., Wang X., Reiss K., Wang S., Malhotra A., Kajstura J., Anversa P. (1998). Stretch-mediated release of angiotensin II induces myocyte apoptosis by activating p53 that enhances the local renin-angiotensin system and decreases the Bcl-2-to-Bax protein ratio in the cell. J. Clin. Investig..

[40] Hsu C.Y. (2006). Antioxidant activity of extract from *Polygonum aviculare* L.. Biol. Res..

[41] Cao J.P., Hu L.J., Li X.L., Xiao H., Tao D.D., Hu J.B., Gong J.P. (2008). Preparation of suspension of gastric mucous membrane single cell and expression of cyclins in cells. Zhonghua Wei Chang Wai Ke Za Zhi.

